# Comparison of intraocular pressure profiles during the water drinking test and the modified diurnal tension curve

**DOI:** 10.1038/s41433-024-02954-0

**Published:** 2024-03-07

**Authors:** Timothy E. Yap, Yuan Gao, Hanif Ahmad, Fernanda Susanna, Remo Susanna, Eduardo M. Normando, Philip A. Bloom, Maria Francesca Cordeiro

**Affiliations:** 1 The Western Eye Hospital, Imperial College Healthcare NHS Trust (ICHNT), London, NW1 5QH UK; 2 The Imperial College Ophthalmic Research Group (ICORG), Imperial College London, London, NW1 5QH UK; 3 Department of Ophthalmology, University of São Paulo, São Paulo, Brazil; 4 Glaucoma and Retinal Neurodegeneration Group, Department of Visual Neuroscience, UCL Institute of Ophthalmology, London, EC1V 9EL UK

**Keywords:** Diagnosis, Biomarkers

## Abstract

**Objectives:**

To compare intraocular pressure (IOP) during the water drinking test (WDT) and modified diurnal tension curve (mDTC) in open-angle glaucoma (OAG) patients, using multimodal, observer-masked tonometry.

**Methods:**

Open-angle glaucoma subjects were prospectively enroled, excluding those who had undergone glaucoma filtration or laser surgery. Two-hourly mDTC Goldmann applanation (GAT) and rebound tonometry (RT) was performed between 8:00 and 16:00, and every 15 min for 45 min after ingestion of 800mls of water. Blood pressure, heart rate, pupillometry measurements, and optical coherence tomography (AS-OCT) were also recorded.

**Results:**

Forty-two subjects’ right eyes were included. 48% were using topical glaucoma medication. Mean baseline IOP was 14.9 ± 4.52 mmHg, with mean visual field mean deviation (±SD) −5.05 ± 5.45 dB. Strong association was found between maximum IOP during mDTC and WDT (*r* = 0.90, 95% CI 0.82–0.95 *p* < 0.0001) with agreement (mDTC-WDT) bias −0.82 mmHg, 95% LoA −1.46 to −0.18. During the WDT, mean systolic blood pressure (±SD) increased from 140.0 ± 20.0 to 153.3 ± 24.0 mmHg (p < 0.0001), mean heart rate ( ± SD) reduced from 69.5 ± 11.3 bpm to 63.6 ± 10.0 bpm (*p* < 0.0001), and temporal iridocorneal angle increased from 29.2 ± 6.0° to 29.6 ± 5.2° (*p* = 0.04).

**Conclusion:**

This study presents repeated, observer-masked IOP data showing strong correlation between maximum IOP during mDTC and WDT using multimodal tonometry. This supports WDT as a meaningful alternative to mDTC when investigating diurnal IOP characteristics in clinic, with reduced time requirements and associated costs.

## Introduction

Despite many large glaucoma trials demonstrating the importance of lowering intraocular pressure (IOP) to prevent glaucoma progression [1, 2], the manner in which we monitor IOP remains clinically inadequate [3]. IOP is routinely measured during working hours, however, maximum IOP is found outside these times in up to 75% of cases [4, 5]. Several methods have been used to detect maximum IOP. The 24-h diurnal monitoring is costly and labour intensive. Continuous contact lens sensors are also time and resource-consuming, may be inaccurate based on corneal curvature, thickness and hysteresis, and don’t estimate IOP in mmHg [6].

The most commonly used investigation for detecting IOP peaks and variation is the modified diurnal tension curve (mDTC), where IOP is measured at 2-hourly intervals throughout the day typically between 8:00 and 16:00. The water drinking test (WDT) has been proposed as an alternative method, whereby IOP is monitored at 15-minute intervals for 45 minutes after drinking 800 mls of water. The maximum IOP during the WDT has previously been correlated with maximum diurnal IOP [7] and progression of glaucoma [8]. Given the potential for time and cost savings, the WDT has been proposed as an alternative to the mDTC to estimate maximum IOP and variability [9, 10].

The mechanism of IOP increase during the WDT in certain individuals remains uncertain, with several hypotheses presented. Choroidal expansion during the WDT has been observed in multiple studies in both glaucomatous [11–13] and non-glaucomatous [14–17] eyes. Reduction in aqueous outflow due to increased episcleral venous pressure [18] has also been considered. Increased aqueous production as a consequence of the sympathetic activation following water drinking is another proposed mechanism [19, 20] that may contribute.

The published WDT literature is focused on the correlation of maximum IOP during the WDT and mDTC [7], whilst also emphasising the potential time and cost savings, and good reproducibility (mean difference 0.47 mmHg, 95% limits of reproducibility −4.24 to +3.30 mmHg) [21]. However, the unique aim of this study is to acquire more robust IOP data using observer-masked, repeated measures with which to analyse further characteristics of resultant IOP profiles, in addition to using multimodal tonometry (applanation and rebound). To characterise autonomic activity during the WDT, several physiological parameters (postural blood pressure, heart rate and pupillometry) are also recorded.

## Materials and methods

### Study design

This was a prospective, cross-sectional, method-comparison study. The study was carried out in accordance with the Declaration of Helsinki.

### Ethical approval

This clinical research was sponsored by Imperial College London and given ethical approval by an HRA Research Ethics Committee (REC), IRAS number 264273.

### Participant recruitment

Participants were recruited from a single centre in London, UK (Western Eye Hospital, Imperial College Healthcare NHS Trust). Potential subjects were given a patient information sheet prior to being asked whether they wish to take part in the study. Consent to take part in the study was given both verbally and in writing.

### Eligibility criteria

Eligible participants were between the ages of 18 to 80 years old with a diagnosis of POAG or NTG in either eye characterised by a glaucomatous optic disc and visual field loss, assessed by a senior glaucoma specialist. VF loss was defined according to the modified Anderson’s criteria [22]. Participants were required to be able to consent to the study, have clear optical media, and a spherical equivalent of +/−10 dioptres. Those with secondary glaucoma including angle-closure glaucoma, pseudoexfoliation syndrome, pigment dispersion syndrome, or neovascular glaucoma were excluded. Furthermore, participants were excluded if they had undergone any glaucoma surgery (filtration or laser surgery), or were diagnosed with any other significant ophthalmic retinal or neurological condition. Participants were excluded with serious cardiac, renal disease or organ failure due to the risk of fluid overload, or those with swallowing difficulties due to structural or neurological oesophageal or gastric disease.

### Baseline characteristics

A medical and ophthalmic history, gender, age, ethnicity, drug history and allergies were recorded at baseline. Height, weight, body mass index (BMI), blood pressure, heart rate, and oxygen saturations were also recorded. Ophthalmic examination included best corrected visual acuity, slit lamp examination, applanation and rebound tonometry, gonioscopy, pachymetry, and pupil examination.

### Automated perimetry

Visual field testing was performed on each eye separately using an automated perimeter (Humphrey Visual Field Analyser 750i, Carl Zeiss Meditech, Jena, Germany) following a 24–2 testing protocol.

### Optical coherence tomography

SD-OCT (Spectralis, Heidelberg Engineering, Heidelberg, Germany) was acquired before and 30 min into the WDT. This included cRNFL thickness (circumpapillary diameter 3.5 mm) and posterior pole macular scans (30° × 25°, 61 B scans each of 768A scans, 120 μm intervals). Anterior segment imaging consisted of imaging the horizontal ICA (30° × 10°, 21 B scans each of 768A scans) and central corneal thickness (CCT) at the corneal apex (15° × 5°, 11 B scans each of 768A scans, 278 μm intervals).

### Intraocular pressure measurements

IOP was measured at 8:00, 10:00, 12:00, 14:00, 16:00 for the mDTC. Following the last IOP measurement, subjects drank 800mls of room temperature water in 5 min. IOP was then measured 15 min, 30 min and 45 min into the WDT. Goldmann applanation tonometry (GAT, Haag-Streit, Koeniz, Switzerland) and rebound tonometry (iCare IC200, Centervue, Vantaa, Finland) were used at each timepoint. GAT readings were measured in a masked fashion with a second practitioner reading and recording IOP measurements. For each timepoint, GAT was carried out twice, and a third measurement was requested by the unmasked practitioner if the first two readings were more than 1 mmHg apart. A mean value was taken in the case of two readings, and a median value taken in the case of three readings to protect against outliers. A mean of six rebound tonometry readings was taken at each timepoint.

Maximum, minimum, integral, mean, standard deviation (SD), and range of IOP was calculated over the five readings taken during the mDTC and the four readings of the WDT. Integral IOP was defined as the area under the IOP graph, in mmHg hours [23, 24]. This was calculated by a custom script in R [25]. An adjustment factor (8 h/0.75 h) was applied to compare GAT and WDT integral IOP.

### Corneal thickness and iridocorneal angle size

CCT and ICA were measured from randomised, anonymised OCT images by two masked assessors (YG and HA) on the same computer under similar lighting conditions. For CCT, a predefined central (X,Y) co-ordinate was chosen with consistent magnification and calliper techniques employed. Bland–Altman analysis highlighted measurements outside the 95% limits of agreement which were repeated by a third masked assessor (TY). For ICA size, a predefined B scan dissecting the horizontal meridian of the anterior chamber was chosen, with consistent magnification and calliper techniques employed measuring between the rise of the peripheral cornea from the trabecular meshwork, and the peripheral iris. Any measurements more than 5° apart were repeated by the third assessor. If readings from two graders were recorded, a mean measurement was used. In cases where a third grader was required to record a measurement, a median was used.

### Pupillometry

Pupillometry was conducted using a handheld automated pupillometry device (NPi-200, NeurOptics, Irvine, USA) in constant mesopic lighting conditions. Minimum pupil diameter, change in pupil size, constriction velocity, maximum constriction velocity, latency and dilation velocity were recorded. Neurological pupil index (NPi^TM^) was calculated by the pupillometer, derived from comparing multiple variables including pupil size, latency, constriction velocity and dilation velocity in reaction to light against a normative database, and given a grading of 0 to 5. An NPi of 3–5 is classified as ‘normal’, equating to an observed ‘brisk’ reaction [26]. Pupil reactions were examined for a relative afferent pupillary defect with a bright handheld light source.

### Blood pressure and heart rate

Heart rate (HR) was measured in a seated position, using the automated readout from the pulse oximeter. Systolic (SBP) and diastolic blood pressure (DBP) was measured using an automated non-invasive blood pressure monitor (Connex Spot Vital Signs Monitor, Welch Allyn, Auburn, USA) fitted around the upper arm in an upright seated position. SBP and DBP readings were repeated after one minute of standing, and the change recorded (postural SBP/DBP change). All HR and BP measurements were acquired immediately prior to water drinking, and 30 min afterwards.

### Statistics

A power calculation was prospectively conducted to give the study 80% power to detect a 2 mmHg difference in IOP maximum (sd 4 mmHg) with ∝ = 5%. Minimum paired sample size was 34 subjects [21]. With around 15% attrition we aimed to recruit at least 40 subjects. The right eye from each subject was included in the analysis. Data analysis was carried out in R [25]. Data distribution was examined for normality using histograms and the Shapiro-Wilk test. Means were compared using a student’s t-test or paired t-test. Correlation was assessed using Pearson’s product-moment correlation [27, 28] and expressed as a correlation coefficient (r). Correlation (r) was deemed very highly positive with r of 0.9–1.0, highly positive between 0.7–0.9, moderate between 0.5–0.7, low positive between 0.3–0.5, and negligible between 0–0.3 [29]. Goodness of fit was measured with the coefficient of determination (R^2^). Paired data was tested for proportional differences using McNemar’s test. Agreement was measured with Cohen’s kappa statistic and Bland–Altman analysis [30]. Logistic regression was used to look for association between predictor variables and binary outcomes. Area under the receiver operating characteristic curves were used to test discriminative ability. P-values were considered significant when less than 0.05 throughout.

## Results

### Subject demographics and baseline characteristics

Forty-two right eyes of 42 eligible subjects met the inclusion criteria for the study, 20 eyes of which (48%) were on anti-ocular hypertensive medication. Demographics and baseline characteristics are displayed in Table 1. A CONSORT diagram in Supplementary Appendix 1 demonstrates participants in each stage of the study.

**Table 1:** The demographics and baseline characteristics of study participants.

Patient demographics	
Patients	42
Eyes	42
Gender (Males : Female)	48%:52%
Ethnicity, n	
Caucasian	22 (52%)
African/Afro-Caribbean	12 (29%)
Asian	6 (14%)
Unknown	2 (5%)
Patient Baseline Characteristics	
Age (Mean ± SD, yrs)	65.5 ± 9.04
BMI (Mean ± SD, kg/m²)	28.9 ± 4.73
Height (Mean ± SD, m)	1.68 ± 0.10
Weight (Mean ± SD, kg)	76.1 ± 16.0
Patients on systemic anti-hypertensive medications, *n*	17 (40%)
Estimated Mean Arterial Pressure (MAP) (Mean ± SD, mmHg)	101.2 ± 12.4
Pulse pressure (Mean ± SD, mmHg)	58.2 ± 17.5
Systolic Blood Pressure (Mean ± SD, mmHg)	140.0 ± 20.0
Diastolic Blood Pressure (Mean ± SD, mmHg)	81.8 ± 11.6
Heart Rate (Mean ± SD, bpm)	69.5 ± 11.3
Oxygen saturations (Mean ± SD, mmHg)	97.5 ± 1.57
Eye Baseline Characteristics	
BCVA (Mean ± SD, LogMAR)	0.12 ± 0.22
Central Corneal Thickness, (Mean ± SD, μm)	542.0 ± 36.3
IOP (Mean ± SD, mmHg)	14.9 ± 4.52
Cup : Disc Ratio	0.66 ± 0.16
Mean global circumpapillary RNFL thickness, μm	76.3 ± 14.3
Mean Deviation (Mean ± SD, dB)	−5.05 ± 5.45
- Mild, MD > −6 dB (Number of eyes)	30 (71%)
- Moderate, −6 dB > MD > −12 dB (Number of eyes)	5 (12%)
- Severe, MD < −12 dB (Number of eyes)	7 (17%)
Pattern Standard Deviation (Mean ± SD, dB)	5.69 ± 4.76
Visual Field Index (Mean ± SD, %)	86.7 ± 15.9
Eyes on anti-ocular hypertensives (Number)	20 (48%)
Temporal angle size (°)	29.1 ± 5.99
Nasal angle size (°)	29.9 ± 5.13
Pupil size (Mean ± SD, mm)	4.37 ± 0.96
Neurological Pupil Index	4.41 ± 0.34

### IOP tonometry method

Across all IOP measurement episodes, there was strongly positive correlation between applanation and rebound tonometry (*r* = 0.89, *p* < 0.0001), with a bias of −0.21 mmHg and 95% limits of agreement ±4.09 mmHg.

### IOP comparisons between mDTC and WDT

A summary of IOP readings is displayed in Table 2. There was a significantly greater mean maximum, integral, minimum, and mean IOP during the WDT when compared to the mDTC (*p* < 0.05).

**Table 2:** Comparison of IOP summary parameters between mDTC and WDT for both applanation and rebound tonometry.

	IOP Characteristics					
	Applanation tonometry			Rebound tonometry		
	mDTC	WDT	*p*	mDTC	WDT	*p*
Maximum IOP (Mean ± SD, mmHg)	16.7 ± 4.38	17.5 ± 4.72	**0.01**	17.2 ± 5.00	18.5 ± 5.46	**0.006**
Off treatment	17.3 ± 4.69	18.6 ± 5.20	**0.01**	17.9 ± 5.44	19.4 ± 6.30	**0.04**
On treatment	16.1 ± 4.04	16.4 ± 3.95	0.46	16.5 ± 4.51	17.5 ± 4.38	0.08
Minimum IOP (Mean ± SD, mmHg)	12.5 ± 3.61	13.4 ± 4.09	**0.006**	12.6 ± 4.09	13.8 ± 4.31	**0.001**
Off treatment	12.6 ± 3.74	13.6 ± 4.49	**0.048**	13.0 ± 4.69	14.5 ± 4.78	**0.007**
On treatment	12.3 ± 3.55	13.1 ± 3.70	**0.04**	12.2 ± 3.39	13.0 ± 3.72	**0.047**
Mean IOP (Mean ± SD, mmHg)	14.6 ± 3.90	15.6 ± 4.43	**0.0008**	14.8 ± 4.28	16.4 ± 5.01	**<0.0001**
Off treatment	51.1 ± 4.12	16.3 ± 4.87	**0.007**	15.3 ± 4.80	17.2 ± 5.92	**0.0003**
On treatment	14.2 ± 3.68	14.8 ± 3.88	0.06	14.3 ± 3.69	15.5 ± 3.80	**0.004**
Integral IOP (Mean ± SD, mmHghrs)	117.4 ± 31.2	127.3 ± 35.6	**<0.0001**	119.0 ± 34.2	134.0 ± 41.2	**<0.0001**
Off treatment	120.7 ± 32.8	133.6 ± 39.1	**0.0007**	122.5 ± 38.4	140.8 ± 48.8	**<0.0001**
On treatment	113.7 ± 29.7	120.3 ± 30.8	**0.04**	114.9 ± 29.3	126.6 ± 30.9	**0.003**
IOP range (Mean ± SD, mmHg)	4.26 ± 1.84	4.18 ± 2.23	0.08	4.63 ± 2.43	4.69 ± 2.32	0.91
Off treatment	4.66 ± 1.94	4.95 ± 2.51	0.63	4.95 ± 2.78	4.86 ± 2.72	0.87
On treatment	3.83 ± 1.66	3.33 ± 1.53	0.36	4.28 ± 2.01	4.51 ± 1.86	0.67
Number over 21 mmHg (*n*)	5 (12%)	8 (19%)		6 (14%)	10 (24%)	
Off treatment	4 (10%)	6 (14%)		4 (10%)	6 (14%)	
On treatment	1 (2%)	2 (5%)		2 (5%)	4 (10%)	

Bold numbers indicate *p* < 0.05.

**Fig. 1:**
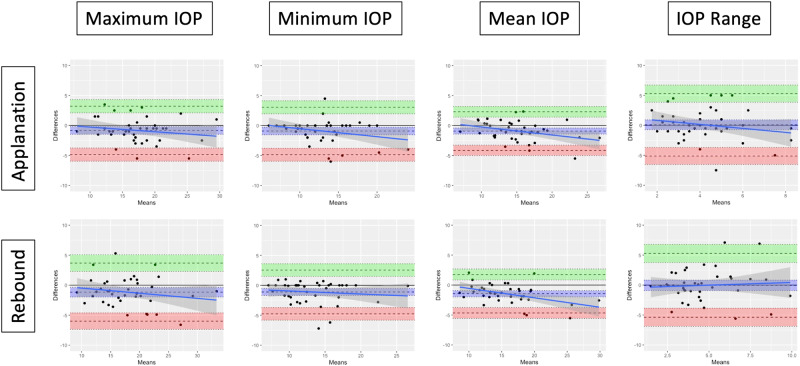
Bland–Altman plots demonstrating the difference in IOP characteristics between mDTC and WDT (mDTC - WDT), using applanation (top row) and rebound (bottom row) tonometry. Mean difference (bias) ± 95% CI, upper 95% limit of agreement ± 95% CI, and the lower 95% limit of agreement ± 95% CI, are displayed (dotted lines) with a regression line ± 95% CI (solid line).

The correlation and agreement of IOP readings between the mDTC and WDT are summarised for applanation tonometry in Table 3, and for rebound tonometry in Appendix 3, with Bland–Altman plots displayed in Fig. 1. For applanation tonometry, significant correlation (*p* < 0.05) was observed between mDTC and WDT for maximum (*r* = 0.9), minimum (*r* = 0.87), mean (*r* = 0.93), and integral IOP (*r* = 0.91), but not for SD or range (*p* > 0.05). For rebound tonometry, significant correlation (*p* < 0.05) was observed between mDTC and WDT for maximum (*r* = 0.89), minimum (*r* = 0.90), mean (*r* = 0.95), integral (*r* = 0.94), SD (*r* = 0.41), and range (*r* = 0.36) of IOP.

**Table 3:** Correlation and agreements statistics of IOP parameters between mDTC and WDT, as measured by applanation tonometry.

	Correlation and Agreement of IOP Characteristics between mDTC and WDT (Applanation tonometry)										
	*r*	95% CI	Est. coefficient	Std.Error	*p*	Bias	95% CI	Lower LoA	95% CI	Upper LoA	95% CI
Maximum IOP (Mean ± SD, mmHg)	0.90	0.82 to 0.95	0.97	0.07	**<0.0001**	−0.82	−1.46 to −0.18	−4.85	−5.96 to −3.75	3.21	2.11 to 4.31
Off treatment	0.90	0.78 to 0.96	1.00	0.11	**<0.0001**	−1.30	−2.28 to −0.31	−5.63	−7.34 to −3.93	3.04	1.34 to 4.74
On treatment	0.90	0.76 to 0.96	0.88	0.10	**<0.0001**	−0.30	−1.13 to 0.53	−3.79	−5.24 to −2.34	3.19	1.74 to 4.64
Minimum IOP (Mean ± SD, mmHg)	0.87	0.77 to 0.93	0.99	0.09	**<0.0001**	−0.90	−1.53 to −0.28	−4.86	−5.95 to −3.78	3.05	1.97 to 4.14
Off treatment	0.87	0.70 to 0.94	1.04	0.13	**<0.0001**	−1.00	−1.99 to −0.01	−5.38	−7.10 to −3.66	3.38	1.66 to 5.10
On treatment	0.88	0.71 to 0.95	0.92	0.12	**<0.0001**	−0.80	−1.64 to 0.04	−4.33	−5.80 to −2.87	2.73	1.27 to 4.20
Mean IOP (Mean ± SD, mmHg)	0.93	0.87 to 0.96	1.06	0.07	**<0.0001**	−0.91	−1.43 to −0.40	−4.14	−5.02 to −3.26	2.31	1.43 to 3.19
Off treatment	0.93	0.84 to 0.97	1.10	0.10	**<0.0001**	−1.18	−1.99 to −0.36	−4.76	−6.16 to −3.35	2.41	1.00 to 3.81
On treatment	0.93	0.83 to 0.97	0.98	0.09	0.09	−0.63	−1.29 to 0.03	−3.39	−4.53 to −2.24	2.13	0.99 to 3.28
IOP range (Mean ± SD, mmHg)	0.16	−0.15 to 0.44	0.19	0.19	0.31	0.08	−0.74 to 0.91	−5.12	−6.55 to −3.69	5.29	3.86 to 6.71
Off treatment	0.19	−0.25 to 0.56	0.24	0.28	0.40	−0.30	−1.57 to 0.97	−5.91	−8.11 to −3.71	5.32	3.11 to 7.52
On treatment	−0.14	−0.55 to 0.33	−0.13	0.22	0.56	0.50	−0.63 to 1.63	−4.22	−6.17 to −2.26	5.22	3.26 to 7.17
SD IOP (Mean ± SD, mmHg)	0.17	−0.14 to 0.45	0.22	0.20	0.27	−0.12	−0.45 to 0.22	−2.2	−2.82 to −1.66	2.0	1.43 to 2.59
Off treatment	0.19	−0.25 to 0.57	0.25	0.29	0.39	−0.32	−0.84 to 0.20	−2.61	−3.51 to −1.71	1.97	1.07 to 2.87
On treatment	−0.11	−0.52 to 0.35	−0.10	0.22	0.66	0.11	−0.34 to 0.56	−1.77	−2.55 to −0.99	1.99	1.21 to 2.77
Integral IOP (Mean ± SD, mmHghrs)	0.91	0.78 to 0.96	0.94	0.10	**<0.0001**	–	–	–	–	–	–
Off treatment	0.92	0.82 to 0.97	1.10	0.10	**<0.0001**	–	–	–	–	–	–
On treatment	0.91	0.76 to 0.96	0.09	0.01	**<0.0001**	–	–	–	–	–	–

Bold numbers indicate *p* < 0.05.

*LoA* limit of agreement, *CI* confidence interval.

Maximum IOP was less than or equal to 1 mmHg difference between the two investigations in 42.9% of eyes, less than or equal to 2 mmHg different in 66.7%, less than or equal to 3 mmHg different in 88.1%, less than or equal to 5 mmHg different in 95.2%, and less than or equal to 6 mmHg different in 100% of eyes. During the mDTC, maximum IOP was most commonly first recorded at the 8am timepoint (35.7%), and less commonly at the 10am (19.0%), 12 pm (16.7%), 2 pm (16.7%), and 6 pm (11.9%) timepoints, as measured with applanation tonometry. During the WDT, maximum IOP was most commonly first recorded at the 15-minute timepoint (45.2%), followed by the 30-min (40.5%), 45-min (9.5%), and baseline (4.8%) timepoints, as measured with applanation tonometry. There was 93% agreement between mDTC and WDT over whether or not patients reached an IOP greater than 21 mmHg (kappa = 0.73 *p* < 0.0001). In all 3 cases of disagreement, the maximum IOP measured during the WDT was greater in magnitude. Individual IOP profiles during the mDTC and WDT are displayed in Supplementary Appendix 2.

### Ophthalmic and physiological changes during the WDT

Ophthalmic and physiological characteristics before and after water drinking are displayed in Table 4. There was a significant rise in SBP and DBP, as well as a reduction in HR and small increase in temporal ICA size after water drinking (*p* < 0.05).

**Table 4:** The difference in physiological and ocular parameters before and during the WDT.

	Timepoints				
	Baseline		30 min		
	Mean	SD	Mean	SD	*p*
Heart Rate (sitting), bpm (Mean ± SD)	69.5	11.3	63.6	10.0	**<0.0001**
Systolic blood pressure, mmHg (Mean ± SD)	140.0	20.0	153.3	24.0	**<0.0001**
Diastolic blood pressure, mmHg (Mean ± SD)	81.8	11.6	86.3	12.0	**0.001**
Postural change in systolic BP, mmHg (Mean ± SD)	2.67	10.3	1.79	10.4	0.64
Postural change in diastolic BP, mmHg (Mean ± SD)	2.71	6.15	3.9	6.93	0.43
Pupil diameter, mm (Mean ± SD)	4.37	0.96	4.25	1.05	0.13
Neurological pupil index (Mean ± SD)	4.41	0.34	4.42	0.33	0.59
Central cornea thickness (CCT), mm (Mean ± SD)	542.5	36.3	547.3	39.0	0.39
Iridocorneal angle - nasal (ICTA), °(Mean ± SD)	29.9	5.1	29.2	4.4	0.66
Iridocorneal angle - temporal (ICTA), °(Mean ± SD)	29.2	6.0	29.6	5.2	**0.04**

Comparison using a paired t-test. Bold numbers indicate *p* < 0.05.

## Discussion

This study provides evidence for a highly positive correlation of maximum IOP during the mDTC and the WDT in open-angle glaucoma patients, including subjects on ocular hypotensive therapy, acquired using repeated, observer-masked data. Associated with rise in mean IOP during the WDT were raised systemic blood pressure, reduction in heart rate, and increase in temporal iridocorneal angle size. A strong agreement between applanation and rebound tonometry was also observed for this application.

These new robust data add to the previously published unmasked studies describing the positive correlation of maximum IOP during the mDTC and WDT including Vasconcelos-Moraes et al. [7] (*r* = 0.78, *n* = 97, 95% CI 0.72–0.83) and Kumar et al. [9] (*r* = 0.88, *n* = 25, 95% CI 0.74–0.94) compared to *r* = 0.90 (95%CI 0.82–0.95) in this study. Although this correlation is in part useful for risk stratification of patients, this does not reflect agreement between the investigations. This study demonstrated 93% agreement on eyes with IOP over 21 mmHg, a mean difference (bias) in maximum IOP of 0.82 mmHg (towards higher IOP in the WDT), and 95% limits of agreement within 4 mmHg. Vasconcelos-Moraes et al. reported greater bias of 2.1 mmHg (towards higher IOP in WDT) with larger 95% limits of agreement (±6 mmHg) which is consistent with their washout from IOP-lowering medications. The bias towards higher IOPs during the WDT may suggest higher IOPs encountered outside of daytime working hours, thus increasing the sensitivity for occult ocular hypertension. Conversely, this may represent artificially elevated IOPs, as a result of water drinking. The former hypothesis is supported by previous publications showing that early morning IOP maxima most commonly occur outside working hours, using both 24-h diurnal tension curves [31–33] and continuous contact lens monitoring [34], and correlate with WDT maxima. As to the relevance of these maximum IOP values, Susanna et al. found associations between higher maximum IOP and laterality of more advanced visual field defects, and progressive visual field defects following WDT, in retrospective analyses [35]. The diurnal timing of maximum IOP levels itself may provide severity or prognostic information, as previously reported amongst treated POAG patients [36].

In addition to maximum IOP, this study was unique in analysing complete IOP profiles. From these, integral IOP (area under the curve) was calculated and compared between the two methods. Integral IOP (measured in mmHg hours) was found to closely correlate between mDTC and WDT (*r* = 0.91). The relevance of this metric is supported by published evidence demonstrating the importance of duration and magnitude of IOP in leading to retinal ganglion cell loss [23, 37] and even potential reversibility [38]. However, it was also interesting to note the lack of correlation in IOP range between the two investigations. This may highlight the lack of multi-dimensionality in using range as a metric, potentially fuelling debate in the literature over the importance of short-term (diurnal) IOP variation as an independent risk factor in glaucoma [39–45]. Furthermore, only poor to fair reproducibility of rnage during mDTC [46] and WDT methods [21, 47] has been shown. Having only been described a few times in pre-clinical work [23, 24], it may be that integral IOP as a multi-dimensional biomarker representing cumulative optic nerve stress is of value, however correlation with functional clinical outcomes is still required.

The increase in systemic arterial blood pressure during the WDT is in keeping with non-ophthalmic studies where parallel rises in plasma noradrenaline were observed, suggesting a sympathetic pressor response [19]. It may be relevant to note that increased blood pressure following water drinking was particularly marked in older subjects and those with primary autonomic failure [19], both proposed as risk factors for glaucoma [48, 49]. Other parameters measured in this and other studies do not seem to account for IOP rises after water drinking. The reduction in heart rate consistently observed during the WDT is presumed to be a homoeostatic vagal baroreceptor reflex to maintain constant mean arterial pressure [50]. Although a significant increase (0.9°) in temporal angle size was observed, this is not conceivably linked to any rise in IOP, although the cause of this is unclear. The similar IOP rise seen during the WDT between open-angle and closed-angle patients suggests the ICA size does not play a significant role [51]. Choroidal expansion has been consistently observed following water drinking [11–17], however a swept-source OCT study did not show an association between IOP rise and choroidal thickness [17] which may implicate a combination of mechanisms behind the observed effect. No other major structural changes in pupil size or central corneal thickness were observed, with changes in corneal hysteresis not observed in previous work [52].

This study demonstrates the highly positive correlation of maximum IOP during the mDTC and the WDT, including subjects on ocular hypotensive therapy (48%). The reason for some eyes not receiving anti-ocular hypertensive treatment was not recorded in this study, however, was likely due to satisfactory IOP levels or patient preference. Topical medication use in this study may be considered a strength for multiple reasons. Firstly, roughly half of the eyes demonstrating an IOP greater than 21 mmHg were already on anti-ocular hypertensive treatment showing that the WDT may be used to demonstrate suboptimal treatment. Furthermore, the multimodal tonometry demonstrated a strong correlation and good agreement between applanation and rebound methods. This may provide further time and cost savings when conducting the WDT without tonometry expertise, or as a screening tool to highlight those patients in whom more extensive IOP investigations are indicated.

A limitation of this study was the single time of day used to perform the investigation for all participants, when in clinical practice this is likely to vary. Prior work has claimed poor levels of maximum IOP reproducibility when conducted at different times on three separate days [47], although it was noted that 80% of differences in maximum IOP values in glaucoma patients, and 77% in normal subjects, were less than 3 mmHg. Other studies have reported reproducible results on consecutive days at the same time (16:00) [46], and over longer time periods between the hours of 13:00 and 17:00 [21], and 14:00 and 16:00 in subjects with pseudoexfoliation [53]. This high reproducibility is important to compare the effect of treatment and IOP peaks during follow-up of patients.

In conclusion, our findings provide robust evidence that the WDT is a meaningful, quicker alternative to the mDTC when attempting to uncover diurnal IOP characteristics in clinic, thus reducing both time requirements and associated costs.

## Summary

### What was known before

- Maximum IOP has previously been shown to correlate between the modified diurnal tension curve and the water drinking test using unmasked application tonometry, and shown to correlate with glaucoma progression.

### What this study adds

- This study provides observer-masked applanation and rebound tonometry data showing that in addition to maximum IOP, there is a strong correlation in minimum, mean, and integral IOP between the modified diurnal tension curve and water drinking tests (WDT), providing potential time and cost savings. A rise in systemic blood pressure and reduction in heart rate during the WDT were observed.

### Supplementary information

Appendix 1

Appendix 2

Appendix 3

Supplemental Material

## Data Availability

The data that support the findings of this study are available from the corresponding author upon reasonable request.
