# Supplementary figures and images for: Comparison of intraocular pressure profiles during the water drinking test and the modified diurnal tension curve
Source: Eye (Lond). 2024 Mar 7;38(8):1567–74. doi: 10.1038/s41433-024-02954-0 (PMC11126618; doi:10.1038/s41433-024-02954-0)

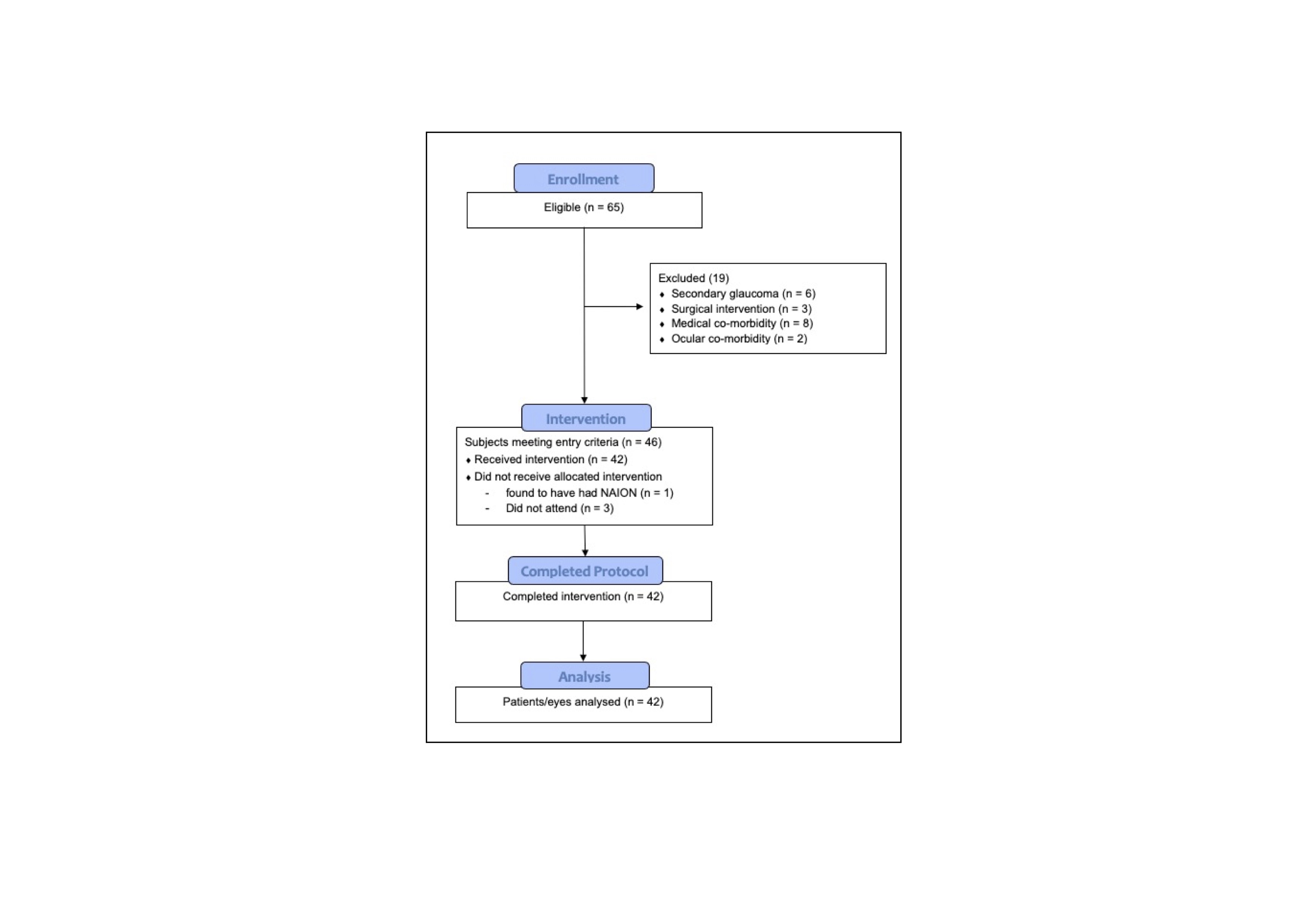

Supplement: Supplementary file 1 — Appendix 1 [file 41433_2024_2954_MOESM1_ESM.jpg]

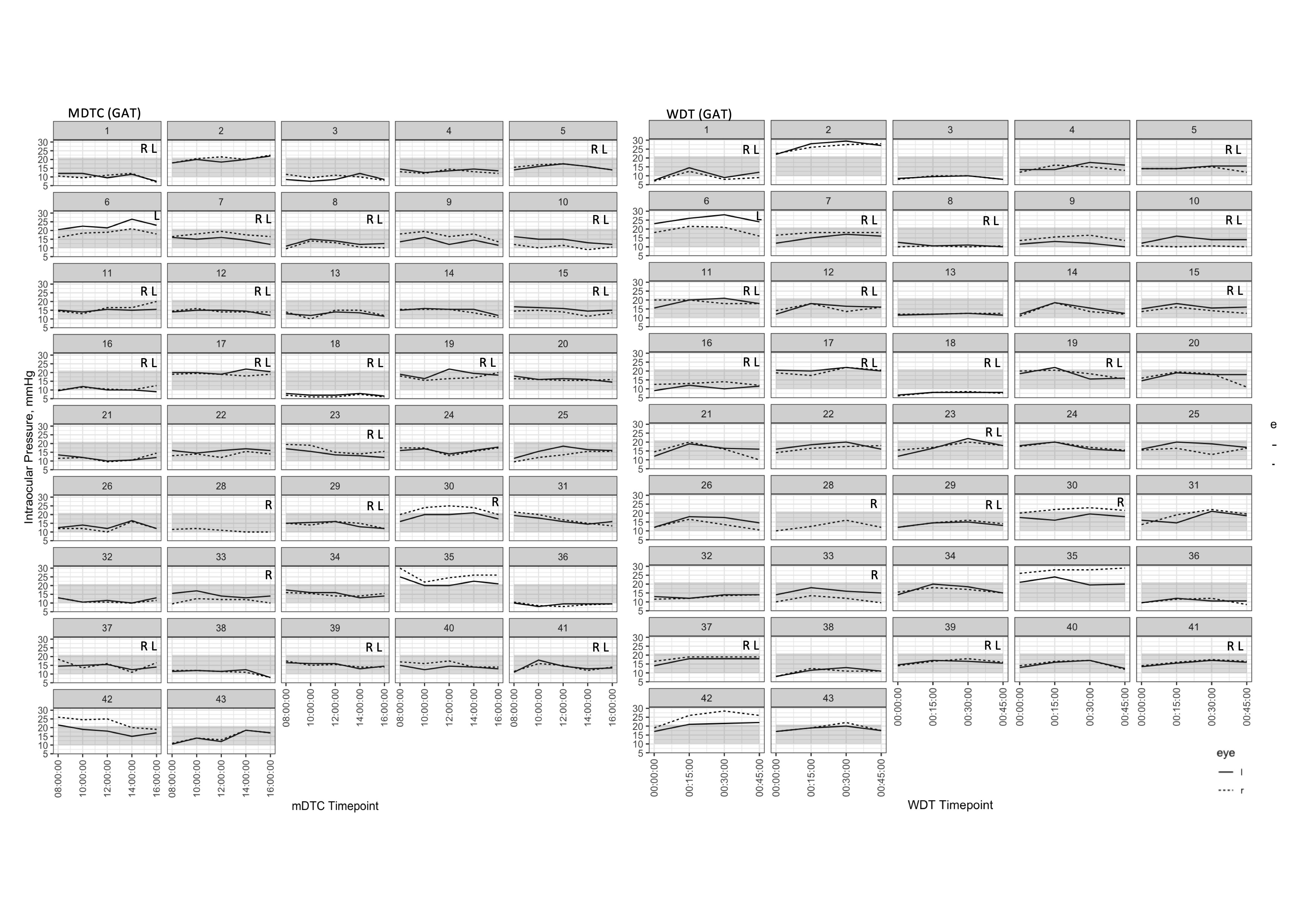

Supplement: Supplementary file 2 — Appendix 2 [file 41433_2024_2954_MOESM2_ESM.jpg]

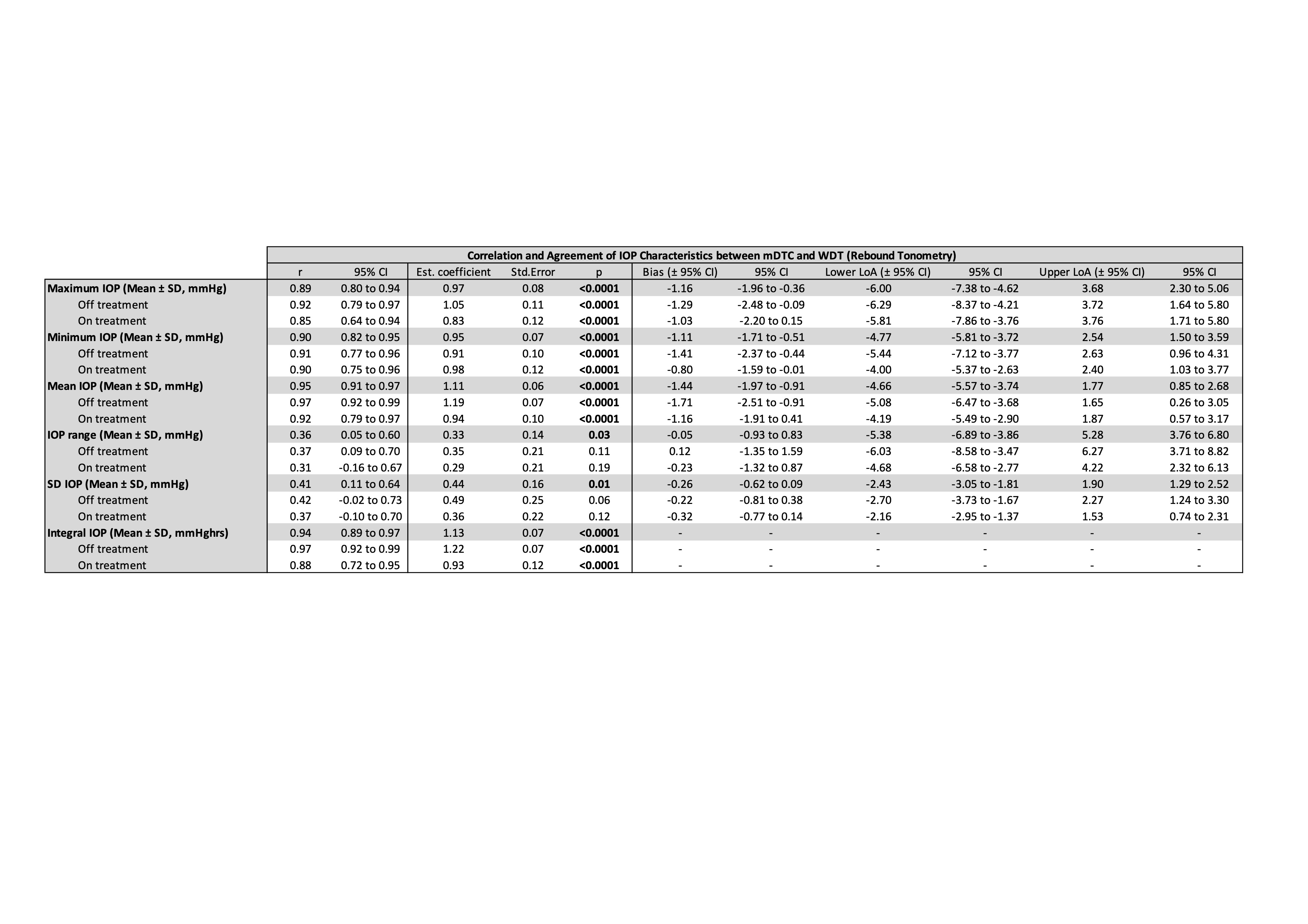

Supplement: Supplementary file 3 — Appendix 3 [file 41433_2024_2954_MOESM3_ESM.jpg]
