# Supplementary material for: Comparison of intraocular pressure profiles during the water drinking test and the modified diurnal tension curve
Source: Eye (Lond). 2024 Mar 7;38(8):1567–74. doi: 10.1038/s41433-024-02954-0 (PMC11126618; doi:10.1038/s41433-024-02954-0)
Supplement: Supplementary file 4 — Supplemental Material [file 41433_2024_2954_MOESM4_ESM.docx]

**Supplemental Material**

***Appendix 1.*** *A CONSORT diagram demonstrating the number of participants in the study. NAION = non-arteritic anterior ischaemic neuropathy.*

***Appendix 2.*** *Intraocular pressure profiles from the right (dotted line) and left (solid line) eyes undergoing mDTC (left panel) and WDT (right panel). Eyes receiving topical anti-ocular hypertensive treatment are indicated with an R (right) or L (left).*

**Appendix 3.** *Correlation and agreements statistics of IOP parameters between mDTC and WDT, as measured by rebound tonometry. Bold numbers indicate p<0.05. LoA = limit of agreement, CI = confidence interval.*
